# Validation of the Gazelle point-of-care device for hemoglobin variant analysis in a predominantly pediatric population: performance comparison with conventional electrophoresis

**DOI:** 10.3389/fmed.2026.1903717

**Published:** 2026-09-22

**Authors:** Rebecca J. Wilson, Kristina Prus, Fatou Ka, Joel E. Mortensen

**Affiliations:** 1 Department of Pathology and Laboratory Medicine, Cincinnati Children’s Hospital, Cincinnati, OH, United States; 2 Department of Pathology and Laboratory Medicine, University of Cincinnati College of Medicine, Cincinnati, OH, United States

**Keywords:** electrophoresis, hemoglobinopathies, point-of-care (POC) diagnosis, sickle cell disease, thalassemia

## Abstract

Sickle cell disease (SCD) is a group of genetic hemoglobinopathies that lead to significant morbidity and mortality including vaso-occlusive crises, hemolysis, and progressive organ damage. Early detection is critical to initiate disease-modifying therapy and prevent complications; however, conventional hemoglobin electrophoresis is often unavailable in low-resource settings where SCD is most prevalent. Point-of-care (POC) devices offer a potential solution by enabling rapid, low-cost screening. The Gazelle POC instrument, which separates hemoglobin variants (A, F, S, and A2/CE) on a cellulose acetate membrane, has been evaluated in limited populations, but its performance relative to conventional electrophoresis methods in pediatric samples remains underexplored. This study validated the analytical and clinical performance of Gazelle using predominantly pediatric whole blood. Precision, linearity, and method comparison studies were conducted with quality control materials and 46 clinical samples analyzed on both Gazelle and the Sebia Capillarys system. Results showed variable precision across hemoglobin variants, strong linearity for Hb A (*R*^2^ ≥ 0.95), and high correlation (*R*^2^ > 0.95) for Hb A, F, and S. These findings support the potential use of Gazelle for SCD screening in children, particularly in resource-limited settings.

## 1 Introduction

Sickle cell disease (SCD) is a group of inherited hemoglobinopathies caused by mutations in the *β*-globin gene which leads to the production of abnormal hemoglobin S (HbS). SCD can cause a spectrum of acute and chronic complications for individuals, ranging from pain crises and anemia to progressive multi-organ damage (1, 2). SCD is a major health concern, affecting 300,000 to 400,000 newborns worldwide, with up to 75% in sub-Saharan Africa (3). Early identification and diagnosis of individuals is crucial for the initiation of prophylactic therapies such as hydroxyurea, which significantly reduce morbidity and mortality (4).

Although hemoglobin electrophoresis and high-performance liquid chromatography (HPLC) remain the gold standards for diagnosis and monitoring of SCD, these methods require sophisticated laboratory infrastructure, trained personnel, and reliable power supply, resources often unavailable in low-income regions where SCD prevalence is highest (5). To overcome these challenges, point-of-care (POC) diagnostic technologies have emerged as promising tools for rapid, low-cost SCD screening (6). One such platform, the Gazelle (Hemex; Portland, United States) device, employs a microchip-based cellulose acetate electrophoresis system to separate and quantify hemoglobin variants (A, F, S, and A₂/CE) within a disposable cartridge (7, 8). Previous validation studies have shown strong agreement between Gazelle and laboratory-based HPLC methods (8).

Currently, the Gazelle device is CE-marked and has been used in India and some African countries as a resource for identifying sickle cell and hemoglobin variants in children and adults (8, 9). This allows patients to be screened in low resource settings without requiring expensive and time-consuming testing. However, limited data are available regarding its analytical performance in pediatric populations and for specific hemoglobin variants, where developmental hemoglobin expression patterns (e.g., elevated Hb F) may influence results. Like some POC devices that detect hemoglobin variants, such as SickleScan, the Gazelle manufacturer does not supply external QC material for testing prior to patient sample, but rather relies on a built-in algorithm that is applied based on internal embedded QC data. Therefore, traditional assessment of between-run precision QC had not necessarily been previously assessed.

This study evaluated the analytical performance of the Gazelle POC device in pediatric and young adult samples. Studies included between-run imprecision using commercial quality control materials, linearity via sample mixing studies, and method correlation with Sebia Capillarys system as a comparator. By generating comprehensive performance data across multiple hemoglobin variants, this research investigated the clinical adoption of Gazelle for SCD screening in children, particularly in resource-limited settings.

## 2 Materials and methods

### 2.1 Device

The Gazelle device is a single-use cartridge-based test that employs a cellulose acetate-based microchip electrophoresis system within a portable reader. A disposable cartridge is used for each sample, and results are reported for four bands: Hemoglobin A, S, F, and A2/CE. Samples are processed according to the manufacturer’s instructions. Briefly, 20 μL of whole blood is mixed with 40 μL of lysing solution. The cellulose acetate membrane inside the Gazelle cartridge is pre-wetted with 50 μL of TBE buffer (pH 8.4), and the lysed blood sample is applied using a sample stamper. Subsequently, 200 μL of TBE buffer is added to both buffer wells, the cartridge is inserted into the Reader, and an electric charge is applied. Separation of hemoglobin bands is completed within 8 min. Interpretation is performed automatically by the device’s integrated software. According to the *Gazelle Hb* Var*iant Physician’s Guide*, version 4, Hb A2, Hb C, and Hb E comigrate within the combined Hb A2/CE region. When beta-thalassemia trait is not detected, the minimum reported Hb A2 value is 2%, and an Hb A2 value greater than 3.6% is used as part of the beta-thalassemia trait interpretation algorithm in patients older than 6 months. The manufacturer reports a 4% limit of detection for Hb A and Hb S; limits of detection for Hb F and Hb A2/CE were not established in the guide. Hb F reporting is phenotype dependent rather than governed by a single universal threshold.

### 2.2 Sample utilization

Samples were selected sequentially from residual patient samples that met predefined inclusion criteria and were analyzed within their established stability windows. The study protocol was reviewed by Cincinnati Children’s Hospital Medical Center Institutional Review Board and approved as expedited (IRB 2024–0069).

### 2.3 Precision

Between-run imprecision was assessed using two patient samples (normal and HbSC patient) and two sets of commercial based quality control (QC) materials: Sickle Chex Positive and Negative controls (Sickle Dex, United States manufacturer), and Bio-Rad Lyphocheck Hemoglobin A2 controls Levels 1 and 2 (Bio-Rad – USA). Bio-Rad Lyphochek Hemoglobin A2 Controls, Level 1 and Level 2 (Product number 553; lots 54,861 and 541,862, respectively), were evaluated. Because these assigned values were method-specific and the commutability of the processed control materials with native whole blood on the Gazelle platform was not established, comparisons with assigned targets were interpreted descriptively and were not considered formal estimates of analytical bias (Supplementary Table S1).

Each commercial QC material was analyzed once daily for 10 consecutive days. The normal and Hb SC residual patient sample was analyzed twice per day for 5 days due to limited sample stability. This design was a preliminary assessment and did not constitute a complete CLSI EP05 precision-establishment study (10). All replicate results were retained in the analysis, and no statistical outliers were excluded. Blank results were treated as missing and were not imputed as 0%. A value of 0% represents the value reported by the Gazelle device and should not be interpreted as independent confirmation that the fraction was analytically absent. For Hb F and Hb A2/CE, device-reported 0% values may also reflect phenotype-dependent reporting or limitations in quantitative assignment. Mean, standard deviation, and coefficient of variation (%CV) were calculated for each fraction. Ninety-five percent confidence intervals for interpretable CV estimates were calculated using Vangel’s modified McKay method. Results below applicable manufacturer-defined reporting thresholds, reported intermittently, or present at or near zero were retained to characterize reporting consistency but were not considered appropriate for quantitative CV interpretation. For these results, detection frequency was reported and the CV was designated as not meaningful.

### 2.4 Linearity

A preliminary linearity assessment adapted from CLSI EP06 was performed using two independent five-level sample-mixing experiments (11). For each experiment, two residual whole-blood specimens representing opposite ends of the evaluated concentration interval were mixed volumetrically at ratios of 100:0, 75:25, 50:50, 25:75, and 0:100. In one of the linearity experiments, the Level 1 source specimen contained mean Gazelle results of 75.5% Hb A2/CE, 24.5% Hb F, 0% Hb S, and 0% Hb A. The Level 5 source specimen contained 3% Hb A2/CE, 0% Hb F, 0% Hb S, and 97% Hb A. In a second linearity experiment, the Level 1 source specimen contained 3% Hb A2/CE, 0% Hb F, 0% Hb S, and 97% Hb A, whereas the Level 5 source specimen contained 0% Hb A2/CE, 17% Hb F, 83% Hb S, and 0% Hb A. The mean duplicate Gazelle results from the two unmixed endpoint specimens were used to assign the endpoint concentrations. Expected concentrations for the intermediate levels were calculated by proportional interpolation according to the volumetric contribution of each source specimen. Each level was analyzed in duplicate, and the individual and mean Gazelle results were compared with the calculated expected concentrations. Concentration-specific deviations from expected values were evaluated, and regression results were reported descriptively. Because expected values were derived from Gazelle measurements and no prospective fraction-specific allowable deviation was defined, the experiments evaluated dilutional proportionality but did not constitute a complete verification of the linearity interval or establish analytical trueness.

### 2.5 Method comparison

Residual whole blood specimens previously analyzed by a reference method, Capillarys 2 Flex Piercing System (Sebia, Inc.), were analyzed on the Gazelle device. The Capillarys hemoglobin(E) assay was performed according to the manufacturer’s procedure and the analytical characteristics described in the FDA 510(k) decision summary (12). Using an alkaline pH buffer, normal and variant hemoglobins were separated and detected in the following order (from cathode to anode): A2’ (A2 variant), C, A2/O-Arab, E, S, D, G-Philadelphia, F, A, Hope, Bart, J, N-Baltimore, and H using Capillarys 2 Flex Piercing System. Residual patient samples were stored refrigerated (2-8C) and tested within 7 days based on manufacturer stability (12). Stability was not independently evaluated on the Gazelle platform in this study. For comparison of the Gazelle Hb A2/CE region with Capillarys results, the Capillarys percentages for Hb A2, Hb C, and Hb E were summed to generate a composite value. This approach was used because Gazelle reports these fractions within a single combined Hb A2/CE region, whereas Capillarys separates and quantifies them individually. The resulting composite was intended to approximate the total proportion of hemoglobin contributing to the Gazelle Hb A2/CE region. This comparison does not establish analytical equivalence for the individual Hb A2, Hb C, or Hb E fractions and was interpreted cautiously in specimens containing multiple or uncommon variants. Because Gazelle applies phenotype-dependent Hb F reporting criteria, a reported Hb *F* value of 0% was interpreted as a device-reported result and not necessarily as confirmation of the analytical absence of Hb F.

Bias was calculated between the methods using Capillarys as the reference method (13). Weighted-Deming regression and Bland–Altman analysis were employed to assess agreement with each hemoglobin variant band (A, A2CE, F, and S). A bias of 20% and/or R^2 of 0.9 was used as acceptance criteria. A sensitivity analysis was performed to evaluate the potential influence of uncommon or co-migrating hemoglobin variants on the quantitative method-comparison results. Specimens containing Hb Bart’s, Hb Inkster, or gamma-chain variants were excluded, and the Deming regression and Bland–Altman analyses were repeated using the remaining complete paired results. Blank or nonnumeric results were treated as missing and were not imputed as zero. Differences for Bland–Altman analysis were calculated as the Gazelle result minus the corresponding Capillarys result.

Phenotype concordance was assessed at three levels. Exact agreement required identical phenotype reporting by Gazelle and Capillarys. Clinically harmonized agreement allowed differences in nomenclature or ordering of the same major hemoglobin fractions when the resulting clinical interpretation was equivalent. Primary-pattern agreement additionally included specimens in which Gazelle identified the major hemoglobin pattern but Capillarys identified an additional secondary finding, such as Hb Bart’s, altered Hb A2, or an uncommon variant; these findings were recorded separately as secondary discordances. Results in which the methods differed in the major hemoglobin pattern or in classification as age-appropriate normal versus potentially abnormal were considered materially discordant. For binary diagnostic performance, reference-positive and reference-negative classifications were assigned using patient age and the manufacturer-relevant interpretation categories for patients ≤6 months and >6 months of age. Sensitivity, specificity, positive predictive value (PPV), negative predictive value (NPV), and overall accuracy were calculated using Capillarys as the comparator, with 95% confidence intervals calculated using the Wilson method. ROC curve analysis and AUC were not performed because the Gazelle phenotype output is categorical and does not provide a continuous diagnostic score across alternative decision thresholds.

### 2.6 Statistical analysis software

Precision, linearity, and method correlation data were analyzed using R Studio (R Foundation, Version 2024.12.1 + 563) and EP Evaluator (Version 11, Data Innovations, Inc.).

## 3 Results

### 3.1 Precision

Table 1 summarizes performance of two sets of commercial QC materials and two patient samples analyzing various Hb variants using mean, standard deviation, and %CV. Each material was analyzed 10 times, and no results were excluded as outliers (Supplementary Tables S2, S3). For fractions consistently detected in all 10 replicates with an interpretable positive mean, 95% confidence intervals for the CV were calculated using Vangel’s modified McKay method.

**Table 1:** Preliminary precision of Gazelle hemoglobin-fraction measurements in commercial quality-control materials and residual patient samples.

Hb fraction	Material	Mean (%)	SD (%)	CV, % (95% CI)	Detected, n/10
Hb A	Sickle-Chex positive	49.3	12.0	24.4 (16.5–47.0)	10/10
	Sickle-Chex negative	61.8	3.7	6.0 (4.1–11.0)	10/10
	Bio-Rad Level 1	77.5	24.2	31.2 (21.0–62.7)	10/10
	Bio-Rad Level 2	54.2	9.5	17.6 (12.0–33.0)	10/10
	Normal patient sample	97.1	0.3	0.3 (0.2–0.6)	10/10
	Hb SC patient sample	0.0	0.0	NA	0/10
Hb A2/CE	Sickle-Chex positive	1.6	5.1	NM	1/10
	Sickle-Chex negative	0.0	0.0	NA	0/10
	Bio-Rad Level 1	1.2	1.5	NM	4/10
	Bio-Rad Level 2	0.0	0.0	NA	0/10
	Normal patient sample	2.9	0.3	10.9 (7.5–20.1)	10/10
	Hb SC patient sample†	57.3	3.3	5.8 (4.0–10.5)	10/10
Hb S	Sickle-Chex positive	39.3	7.4	18.8 (12.8–35.5)	10/10
	Sickle-Chex negative	0.0	0.0	NA	0/10
	Bio-Rad Level 1	1.1	3.5	NM	1/10
	Bio-Rad Level 2	38.8	6.8	17.4 (11.9–32.7)	10/10
	Normal patient sample	0.0	0.0	NA	0/10
	Hb SC patient sample	41.1	2.6	6.4 (4.4–11.8)	10/10
Hb F	Sickle-Chex positive	9.8	15.9	NM	3/10
	Sickle-Chex negative	38.2	3.7	9.7 (6.7–17.9)	10/10
	Bio-Rad Level 1	20.2	21.9	NM	6/10
	Bio-Rad Level 2	7.0	14.8	NM	2/10
	Normal patient sample	0.0	0.0	NA	0/10
	Hb SC patient sample	1.6	2.1	NM	4/10

Mean values, standard deviations (SD), and coefficients of variation (CV) were calculated from replicate analyses of each QC material (Sickle Chek Positive, Sickle Chek Negative, Bio-Rad Level 1, and Bio-Rad Level 2) as well as a normal patient sample and an Hb SC patient sample. Results are presented for Hb A, Hb A2CE, Hb S, and Hb F to illustrate fraction-specific variability across materials with differing hemoglobin compositions.

Each material was evaluated using 10 replicate measurements. Mean and sample standard deviation were calculated using all results; no outliers were excluded. For fractions consistently detected in all 10 replicates with a positive mean and an interpretable CV, two-sided 95% confidence intervals for the CV were calculated using Vangel’s modified McKay method.

CI, confidence interval; CV, coefficient of variation; Hb, hemoglobin; NA, not applicable; NM, not meaningful for quantitative precision interpretation; SD, standard deviation.

NA indicates that the fraction was not detected in any replicate and the CV was undefined. NM indicates that the fraction was intermittently detected, had a mean near zero, or was below the manufacturer-reported quantitative range; therefore, a numerical CV and confidence interval were not considered analytically meaningful. A result of 0% indicates that Gazelle did not report a measurable band and does not represent independent confirmation of an absolute concentration of zero.

†In the Hb SC patient sample, the fraction reported in the Hb A2/CE region predominantly represents Hb C rather than Hb A2 because Gazelle reports Hb A2, Hb C, and Hb E within a combined analytical region.

In terms of commercial QC, Sickle-Chex Negative was the most precise for Hb A, with a CV of 6.0% (95% CI, 4.1–11.0%), whereas Bio-Rad Level 1 had the greatest variability, with a CV of 31.2% (95% CI, 21.0–62.7%). Bio-Rad Level 2 and Sickle-Chex Positive had Hb A CVs of 17.6 and 24.4%, respectively Table 1.

Hb A2/CE was inconsistently reported in the commercial QC materials. It was detected in 1 of 10 Sickle-Chex Positive replicates and 4 of 10 Bio-Rad Level 1 replicates, with mean concentrations of 1.6 and 1.2%, respectively. These concentrations were below the manufacturer-described minimum Hb A2 reporting value of 2%, and the CVs were therefore not considered meaningful for quantitative interpretation. Hb A2/CE was not reported in Sickle-Chex Negative or Bio-Rad Level 2. Because Gazelle combines Hb A2, Hb C, and Hb E within a single analytical region, these results represent reporting within the composite Hb A2/CE band rather than independent measurement of Hb A2.

For Hb S, Sickle-Chex Positive and Bio-Rad Level 2 demonstrated similar precision, with CVs of 18.8% (95% CI, 12.8–35.5%) and 17.4% (95% CI, 11.9–32.7%), respectively. Bio-Rad Level 1 had a low mean Hb S concentration of 1.1%, with Hb S reported in only 1 of 10 replicates; therefore, a numerical CV was not considered meaningful. Hb S was not reported in Sickle-Chex Negative.

For Hb F, Sickle-Chex Negative demonstrated the lowest imprecision, with a CV of 9.7% (95% CI, 6.7–17.9%) and detection in all 10 replicates. In contrast, Hb F was intermittently reported in Sickle-Chex Positive, Bio-Rad Level 1, and Bio-Rad Level 2, with detection in 3 of 10, 6 of 10, and 2 of 10 replicates, respectively. Because of this intermittent reporting, numerical CVs for these materials were not considered meaningful for quantitative interpretation.

When compared with commercial QC materials, patient samples demonstrated markedly lower variability for hemoglobin fractions present at physiologic or disease-appropriate levels (i.e., SC disease). In the normal patient sample, Hb A and Hb A2CE showed minimum variation (CV 0 and 11%, respectively). Similarly, the Hb SC patient sample showed stable measurements for the expected fraction with a %CV of 8 and 6% for Hb S and Hb A2CE fractions, respectively, while fractions absent or present at trace levels (Hb A and HbF) exhibited higher CVs. In the Hb SC patient sample, the Gazelle Hb A2/CE band predominantly represents Hb C rather than Hb A2.

### 3.2 Linearity

Results from the first mixing experiment are presented in Figure 1, and results from the second experiment are provided in Supplementary Figure S1. For each concentration level, the duplicate Gazelle measurements were averaged. The mean Gazelle results from the two unmixed endpoint specimens, Levels 1 and 5, were used to calculate the expected concentrations of the intermediate levels according to the volumetric mixing ratios. Absolute and percent deviations were calculated by comparing the mean measured Gazelle result with the calculated expected concentration. Linear regression and *R*^2^ were reported descriptively and interpreted together with the concentration-specific deviations rather than using *R*^2^ alone as a criterion for acceptable linearity.

**Figure 1:**
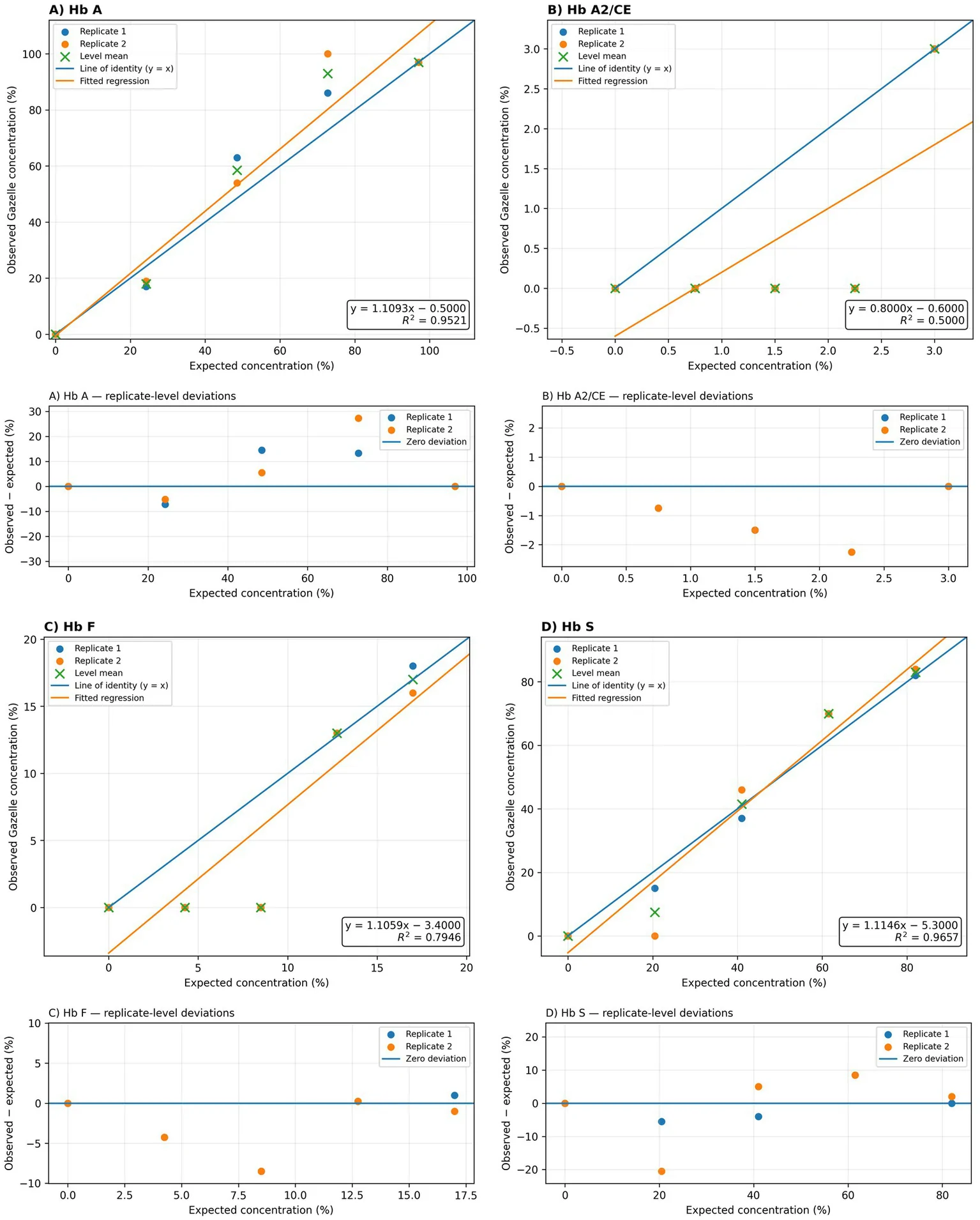
Linearity assessment of hemoglobin fractions of High Hb S and normal sample. Expected versus measured values are shown for (**A**) **Hb A**, (**B**) **Hb A2/E**, (**C**) **Hb F**, and (**D**) **Hb S** using a sample mixing design. Linear regression equations and coefficients of determination (R²) are shown for each analyte.

In the first experiment, a normal specimen was mixed with a high-Hb S specimen. Hb A and Hb S demonstrated stronger linear associations than Hb F and Hb A2/CE, with *R*^2^ values of 0.9521 and 0.9657, respectively (Supplementary Table S4). However, concentration-specific deviations remained evident. For Hb A, expected concentrations of 72.75 and 48.50% produced mean Gazelle results of 93.0 and 58.5%, respectively. For Hb S, the lowest nonzero expected concentration of 20.75% produced duplicate results of 15 and 0%, with a mean of 7.5%. At higher expected Hb S concentrations of 41.50, 62.25, and 83.00%, mean Gazelle results were 41.5, 70.0, and 83.0%, respectively.

Hb F and Hb A2/CE demonstrated weaker concentration-response relationships in the first experiment, with R^2^ values of 0.7946 and 0.5000, respectively. Hb F was not reported in either replicate at expected concentrations of 4.25 and 8.50%, but mean results of 13.0 and 17.0% were observed at expected concentrations of 12.75 and 17.00%, respectively. Hb A2/CE was reported at 3.0% in the unmixed normal specimen but was not reported at calculated expected concentrations of 2.25, 1.50%, or 0.75%.

In the second experiment, a specimen with high Hb A2/CE and Hb F was mixed with a predominantly normal specimen (Supplementary Table S5). Hb A again demonstrated a strong linear association, with an *R*^2^ of 0.9468, although positive deviations were observed at several intermediate levels. Expected Hb A concentrations of 24.25, 48.50, and 72.75% produced mean Gazelle results of 28.0, 66.0, and 94.0%, respectively. Hb F produced an *R*^2^ of 0.8355 and showed concentration-dependent deviations, including a mean result of 19.0% at an expected concentration of 12.25% and no reported Hb F at an expected concentration of 6.125%.

The Hb A2/CE region produced an *R*^2^ of 0.8939 in the second experiment but demonstrated substantial under-recovery at the intermediate levels. Mean Gazelle results were 55.5, 15.0, and 9.0% at expected concentrations of 57.375, 39.25, and 21.125%, respectively. Hb S was absent from both source specimens and all prepared mixtures in the second experiment and therefore was not evaluated. Overall, the two experiments demonstrated stronger proportional responses for Hb A and Hb S than for Hb F and Hb A2/CE, although the high *R*^2^ values for Hb A and Hb S did not exclude clinically relevant concentration-specific deviations.

### 3.3 Method comparison

Method-comparison studies were conducted using 46 specimens with a range of hemoglobin profiles (see, Figure 2 and Figure 3). Individuals ranged in age from 39 days to 48 years, with a mean age of 7.7 years. Forty-one specimens were from pediatric individuals (<18 years) and five were from adults; 17 were from female individuals and 29 from male individuals (Supplementary Tables S6, S7). The Capillarys and Gazelle phenotype distributions are summarized in Supplementary Tables S8, S9. The cohort included uncommon or co-migrating findings including Hb Bart’s, gamma-chain variants, Hb Inkster, and Hb Toulon.

**Figure 2:**
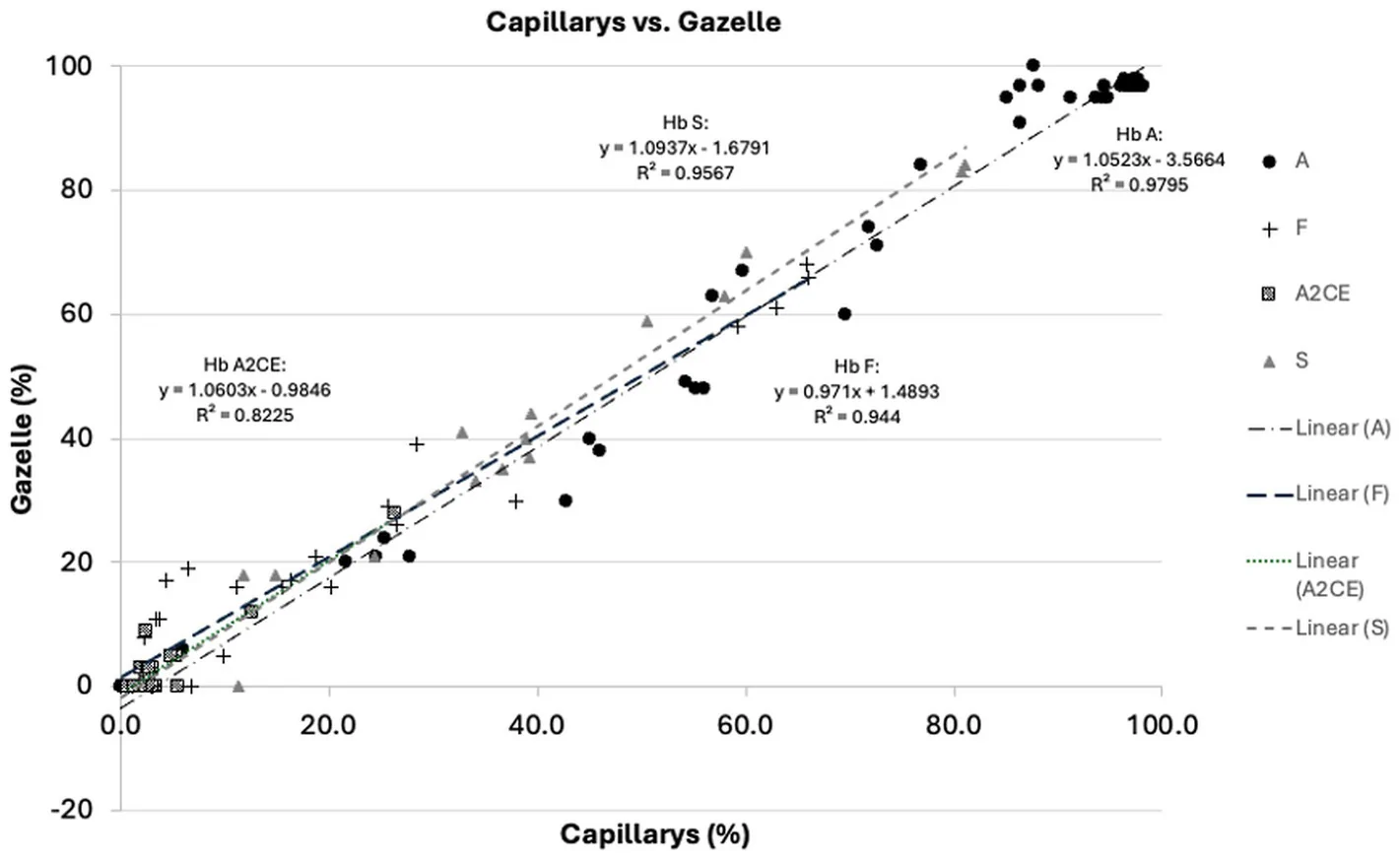
Method correlation of residual whole blood samples between Sebia Capillarys and Gazelle device. Hb fractions are plotted individually with linear regressions applied.

**Figure 3:**
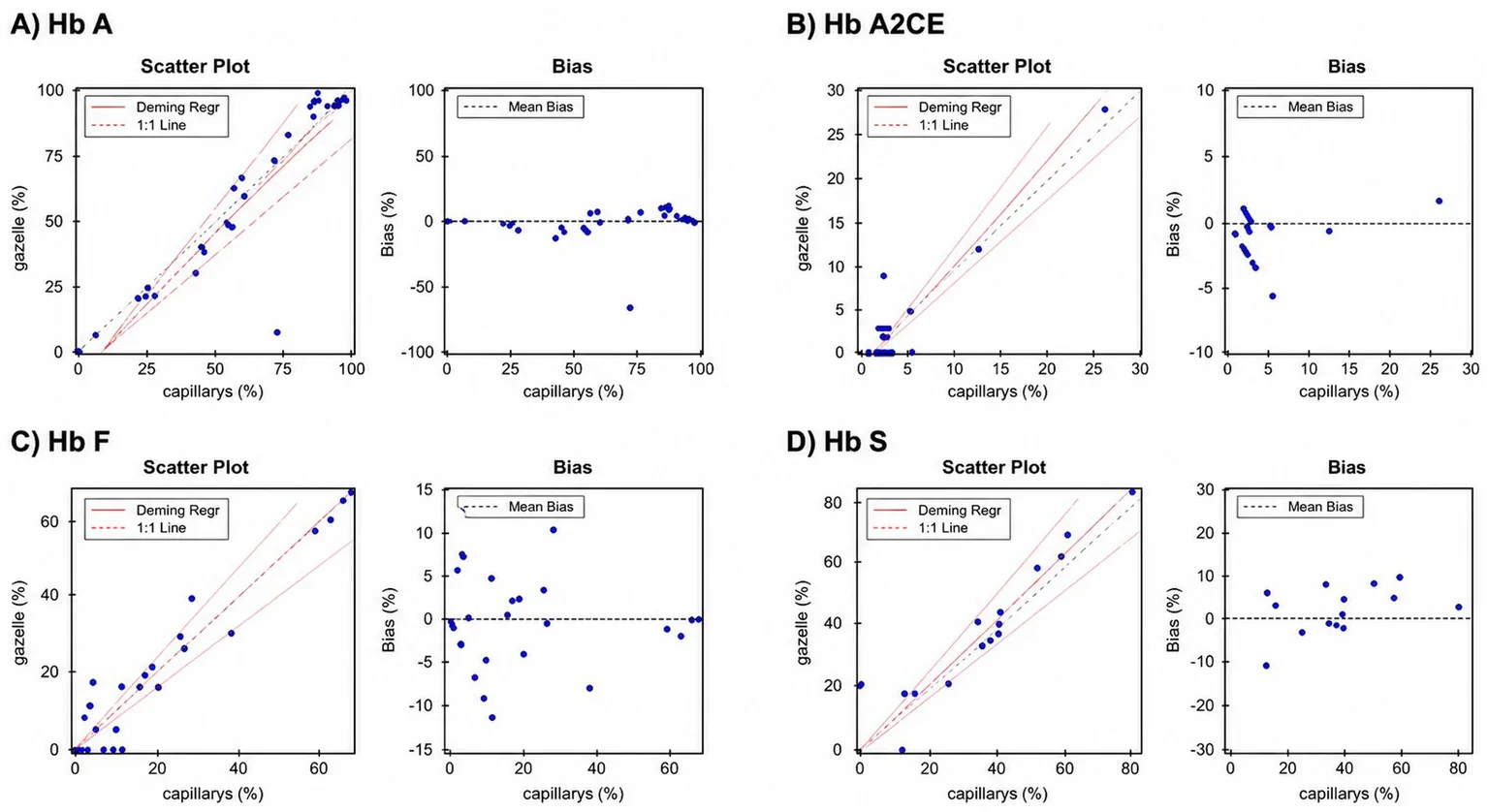
Method comparison of hemoglobin fraction measurements. Comparison of capillary-based testing versus the reference method for (**A**) **Hb A**, (**B**) **Hb A2/E**, (**C**) **Hb F**, and (**D**) **Hb S**. For each analyte, scatter plots (left) display Deming regression (solid line) and the line of identity (dashed line). Corresponding Bland-Altman plots (right) show percent bias versus mean concentration, with mean bias (solid horizontal line) and limits of agreement (+/- 1.96 SD); dashed lines).

Phenotype concordance was evaluated at three levels. Exact phenotype agreement was observed in 21 of 46 specimens (45.7%; 95% CI, 32.2–59.8%). After harmonizing differences in nomenclature or fraction ordering that did not alter the major clinical phenotype, 33 of 46 specimens were concordant (71.7%; 95% CI, 57.5–82.7%). When specimens with the same primary hemoglobin pattern but an additional secondary finding detected by Capillarys were also considered to have primary-pattern agreement, concordance increased to 39 of 46 specimens (84.8%; 95% CI, 71.8–92.4%); seven specimens (15.2%) were materially discordant. Complete paired phenotype classifications and the secondary or materially discordant cases are provided in Supplementary Tables S10, S11. In the age-appropriate binary analysis of abnormal versus age-appropriate normal patterns, sensitivity was 70.0% (21/30), specificity 100% (16/16), PPV 100% (21/21), NPV 64.0% (16/25), and overall accuracy 80.4% (37/46) (Supplementary Table S12). PPV and NPV are specific to this enriched study cohort and should not be generalized to a population-screening prevalence.

For quantitative method comparison, Capillarys Hb A2, Hb C, and Hb E values were summed to create the composite comparator corresponding to the combined Gazelle Hb A2/CE region. For Hb A (*n* = 46), Deming regression yielded a slope of 1.064 and intercept of −4.395, with a mean bias of +0.15 percentage points and 95% limits of agreement of −9.94 to +10.25. For Hb F (32 complete numeric pairs), the slope was 0.999 and intercept +1.040, with a mean bias of +1.03 percentage points and limits of agreement of −8.70 to +10.76. For Hb A2/CE (43 complete numeric pairs), the slope was 1.182 and intercept −1.351, with a mean bias of −0.72 percentage points and limits of agreement of −4.56 to +3.11. For Hb S (15 complete pairs), the slope was 1.121 and intercept −2.794, with a mean bias of +2.15 percentage points and limits of agreement of −8.65 to +12.96. Complete regression and Bland–Altman results are provided in Supplementary Table S14, and the complete paired quantitative dataset is provided in Supplementary Table S16. Hb F was measurable by Capillarys but reported as 0% by Gazelle in 13 of 32 complete numeric pairs. Ten of these specimens had Capillarys Hb F concentrations of 0.4–3.1%, whereas three had concentrations of 6.8, 9.2, and 11.4% (Supplementary Table S13), indicating that low Hb F concentration alone did not account for all nondetections.

Sensitivity analyses excluding specimens containing Hb Bart’s, Hb Inkster, or gamma-chain variants produced results similar to the analyses including all specimens. The Hb A slope changed from 1.064 to 1.056, the Hb F slope from 0.999 to 0.998, and the Hb A2/CE slope from 1.182 to 1.176. Mean biases changed by no more than 0.25 percentage points, and the limits of agreement remained similar. The Hb S analysis was unchanged because the excluded specimens did not contribute complete paired Hb S results. Full estimates with and without uncommon variants are provided in Supplementary Table S14, and the magnitude of change is summarized in Supplementary Table S15. Thus, uncommon variants did not materially affect the aggregate quantitative method-comparison estimates, although they remained important sources of qualitative phenotype discordance.

## 4 Discussion

The present study evaluated the analytical performance of the Gazelle POC device for hemoglobin variant detection in a predominantly pediatric population, with a focus on precision, linearity, and agreement with a capillary electrophoresis reference method. Overall, performance varied by hemoglobin fraction, with the strongest analytical performance observed for Hb A and Hb S, and reduced performance for Hb F and Hb A2/CE, particularly at low concentrations.

When considered alongside prior studies of the Gazelle platform, these findings are not entirely unexpected. Earlier work has demonstrated that the device performs well for identifying major hemoglobin phenotypes, although these studies have largely focused on classification rather than detailed analytical performance (7, 8). By examining individual fractions more closely, it becomes clear that agreement depends not only on the specific hemoglobin variant but also on its relative abundance. Hb A, Hb S, and, to a large extent, Hb F tracked closely with the reference method, whereas Hb A2/CE showed more variable performance, particularly near the lower end of the measurement range. Interpretation of the Hb A2/CE results requires particular caution because Gazelle reports Hb A2, Hb C, and Hb E within a single combined analytical region. To approximate this measurand, the corresponding Capillarys fractions were summed for quantitative comparison. However, agreement between the summed Capillarys value and the Gazelle Hb A2/CE result does not demonstrate accurate identification or quantification of the individual fractions. In samples containing multiple hemoglobin variants or uncommon fractions that migrate within or near this region, summation may obscure clinically important differences in variant identification and may overestimate agreement between the methods. Therefore, the Hb A2/CE analysis should be interpreted as a comparison of the total proportion assigned to a composite migration region rather than as validation of Gazelle for independent Hb A2, Hb C, or Hb E measurement. This distinction between detection and quantitation is important, as the device may identify major hemoglobin patterns while providing less reliable characterization of minor or uncommon fractions.

A similar pattern was observed in the precision data. Variability increased substantially for fractions present at low levels, particularly Hb F and Hb A2/CE, while fractions present at higher concentrations were measured more consistently. This is consistent with expected analytical behavior, as measurements near the limit of detection are inherently more susceptible to variability (14). In contrast, Hb S, which is typically present at higher levels in affected individuals, demonstrated relatively stable performance across both patient samples and quality control materials. Low fraction concentration did not fully explain the observed imprecision. Several processed control materials demonstrated substantial variability for fractions present at moderate or high concentrations. Potential contributors include differences between commercial control matrices and native whole blood, sample reconstitution and application, cartridge-to-cartridge variability, electrophoretic band separation, and automated band recognition or integration. Because these factors were not independently evaluated, their relative contributions could not be determined. Taken together, these findings indicate that imprecision was dependent on both the hemoglobin fraction and the material evaluated and could not be explained solely by analyte concentration. An additional aspect of this study was the use of commercial control materials and residual patient specimens to characterize between-run precision of individual hemoglobin fractions. Gazelle incorporates marker fluid as an internal control for each test, and the manufacturer identifies Sickle-Chex as an external control option. The substantial differences observed among commercial control materials and native patient specimens suggest that material-specific effects should be considered when external controls are used to evaluate Gazelle performance.

Although regression analyses demonstrated relatively strong associations for several hemoglobin fractions, correlation alone does not establish analytical agreement. Bland–Altman analysis showed that relatively small mean biases at the aggregate level could coexist with wider limits of agreement and substantial differences in individual specimens. This was particularly relevant for Hb F and Hb S, where sample-level differences were greater than suggested by the overall regression relationships. These findings emphasize that method performance should be interpreted using both regression and difference analyses rather than correlation alone. Hb F reporting and quantitative agreement varied across concentrations, with several low-concentration fractions not reported by Gazelle. Potential contributors include limited electrophoretic separation of adjacent fractions and automated band recognition or integration, particularly in specimens containing multiple hemoglobin fractions. This reduced separation may impact quantitation in samples where both fractions are elevated. In addition, uncommon variants, such as Hb Barts or Hb Inkster, were observed to migrate within the S or F regions, highlighting a limitation of the device and reinforcing that variant interference remains a consideration even with traditional electrophoretic methods. The manufacturer’s guide includes Hb Bart’s within the infant interpretation algorithm; therefore, patient age and the applicable Gazelle algorithm should be considered when evaluating concordance in Hb Bart’s-containing specimens. Assignment of Hb Bart’s within an Hb F-containing region could be clinically important in neonatal samples, in which Hb F is normally predominant and Hb Bart’s may indicate alpha-globin abnormalities. Similarly, assignment of an uncommon variant such as Hb Inkster to an Hb S or Hb F-containing pattern could produce an incorrect presumptive phenotype. These findings reinforce the need for confirmatory testing when a Gazelle result is abnormal, unexpected, or inconsistent with the clinical context.

Exclusion of specimens containing Hb Bart’s, Hb Inkster, or gamma-chain variants did not materially change the regression slopes, intercepts, mean biases, or limits of agreement. Therefore, these specimens were not the primary explanation for the aggregate quantitative differences between Gazelle and Capillarys. Nevertheless, their qualitative misclassification remains clinically important. Aggregate regression statistics may appear stable even when individual uncommon variants are assigned to an incorrect Gazelle reporting region, reinforcing the need to evaluate phenotype concordance and individual discrepant specimens separately from quantitative method agreement.

When compared with conventional laboratory methods such as HPLC and capillary electrophoresis, the performance characteristics observed here reflect the expected tradeoffs between analytical precision and accessibility. Reference methods are designed to provide highly accurate and reproducible quantitation across a wide range of concentrations, including minor fractions, with coefficients of variation typically below 3–5% (15, 16). In contrast, POC devices prioritize speed, portability, and ease of use. As a result, reduced precision at lower concentrations is not unexpected.

The Hb A2/CE region demonstrated important limitations across both low and higher concentrations. At low concentrations, the fraction was intermittently or not reported, consistent with limitations in quantifying small fractions within the combined Hb A2/CE region. However, the second mixing experiment also demonstrated substantial under-recovery at intermediate expected concentrations, indicating that low concentration alone did not explain the observed discrepancies. Interpretation is further complicated by the fact that Hb A2, Hb C, and Hb E comigrate within a single Gazelle reporting region. Accordingly, agreement in the total Hb A2/CE percentage should not be interpreted as evidence that the device can independently identify or accurately quantify each contributing fraction.

The two preliminary sample-mixing studies also demonstrated fraction-dependent performance. Hb A and Hb S showed stronger proportional relationships between calculated expected and observed concentrations than Hb F and Hb A2/CE; however, concentration-specific deviations were evident even when regression coefficients were relatively high. Hb F showed intermittent reporting at lower expected concentrations, whereas Hb A2/CE demonstrated substantial deviations at both low and intermediate concentrations. These findings emphasize that regression coefficients alone do not establish acceptable quantitative performance across the evaluated interval and further support the use of Gazelle primarily for preliminary screening rather than precise quantification of individual hemoglobin fractions.

One strength of this study is the inclusion of a pediatric population, in which elevated Hb F levels are common and may challenge analytical performance. The predominantly pediatric population is particularly relevant because Hb F concentrations and expected hemoglobin patterns vary substantially with age. Gazelle applies separate interpretation algorithms for patients 6 months of age or younger and those older than 6 months, which should be considered when interpreting phenotype concordance in infants. The results support prior observations that Gazelle can reliably distinguish clinically relevant phenotypes such as Hb SS, Hb SC, and Hb AS. Additionally, this study is among the first to evaluate external QC materials for assessing precision of individual hemoglobin fractions on this platform, providing a practical framework for laboratories seeking to monitor device performance outside of the internal QC system.

Several limitations should be considered. The precision study included only 10 measurements per material and therefore did not fully characterize longer-term, between-operator, or between-lot imprecision. The resulting CV estimates should be interpreted as preliminary. The linearity assessment was also preliminary, with only duplicate measurements at five concentration levels. Expected intermediate concentrations were calculated from Gazelle measurements of the two endpoint specimens rather than independently assigned reference-method values; therefore, these experiments evaluated dilutional proportionality and did not establish analytical trueness or a validated linearity interval. The number of samples containing certain variants, particularly Hb C and Hb E, was limited, restricting evaluation in these populations. The use of residual samples may not fully reflect prospective clinical performance, and longitudinal assessment was not performed. The manufacturer describes Hb F quantification as potentially useful for clinical decision-making during hydroxyurea therapy (17); however, quantitative monitoring was not evaluated in this study. The phenotype-dependent Hb F reporting rules and observed variability limit conclusions regarding serial assessment of disease-modifying therapy. Future studies should include larger and more diverse populations, as well as evaluation of the device in monitoring settings, such as patients undergoing hydroxyurea therapy, where Hb S and Hb F levels may change over time.

Overall, Gazelle may provide a useful screening method for major hemoglobin patterns, particularly in settings where conventional laboratory testing is not immediately available. However, limitations in Hb F detection and reporting, interpretation of the composite Hb A2/CE region, and identification of uncommon variants indicate that abnormal, unexpected, or clinically consequential results require confirmation using an established laboratory method.

## Data Availability

The raw data supporting the conclusions of this article will be made available by the authors, without undue reservation.
