# Supplementary material for: Validation of the Gazelle point-of-care device for hemoglobin variant analysis in a predominantly pediatric population: performance comparison with conventional electrophoresis
Source: Front Med (Lausanne). 2026 Sep 22;13:1903717. doi: 10.3389/fmed.2026.1903717 (PMC13638383; doi:10.3389/fmed.2026.1903717)
Supplement: Supplementary file 1 [file Supplementary_file_1.docx]

**Supplementary Tables**

*Validation of the Gazelle Point-of-Care Device for Hemoglobin Variant Analysis in a Predominantly Pediatric Population: Performance Comparison with Conventional Electrophoresis*

**Supplementary Table S1.** Bio-Rad Lyphochek Hemoglobin A2 Control manufacturer-assigned values for the Sebia CAPILLARYS 2 FLEX-PIERCING system (reference method) and observed Gazelle results.

| **Fraction** | **Control level** | **Manufacturer-assigned mean** | **Assigned range** | **Gazelle mean** | **Difference from assigned mean** |
| --- | --- | --- | --- | --- | --- |
| Hb A | Level 1 | Not provided | N/A | 77.5% | N/A |
| Hb A | Level 2 | Not provided | N/A | 54.2% | N/A |
| Hb A2 | Level 1 | 2.60% | 2.10%–3.10% | 1.2% | −53.8% |
| Hb A2 | Level 2 | 5.50% | 4.50%–6.50% | 0.0% | −100% |
| Hb F | Level 1 | 1.00% | 0.70%–1.30% | 20.2% | +1920% |
| Hb F | Level 2 | 10.0% | 9.00%–11.00% | 7.0% | −30.0% |
| Hb S | Level 1 | None detected | N/A | 1.1% | N/A |
| Hb S | Level 2 | 29.7% | 27.7%–31.7% | 38.8% | +30.6% |

Abbreviations: Hb, hemoglobin; N/A, not applicable. Difference from assigned mean was calculated as [(Gazelle mean − manufacturer-assigned mean) / manufacturer-assigned mean] × 100. Manufacturer-assigned values and ranges are specific to the Sebia CAPILLARYS 2 FLEX-PIERCING system. Because commutability of the processed control material with native whole blood on the Gazelle platform was not established, comparisons with assigned targets are descriptive and should not be interpreted as formal estimates of analytical bias.

**Supplementary Table S2.** Raw Gazelle hemoglobin-fraction results for commercial quality-control materials.

Each commercial quality-control material was analyzed once daily for 10 runs. Results represent the percentage of total hemoglobin assigned by the Gazelle device to the Hb A, Hb A2/CE, Hb F, and Hb S bands. A value of 0 indicates that the device did not report a measurable band in that region; it should not be interpreted as an independently confirmed concentration of absolute zero. All replicate results are shown, and no results were excluded as outliers.

**A. Sickle-Chex Positive**

| **Gazelle band** | **Run 1** | **Run 2** | **Run 3** | **Run 4** | **Run 5** | **Run 6** | **Run 7** | **Run 8** | **Run 9** | **Run 10** |
| --- | --- | --- | --- | --- | --- | --- | --- | --- | --- | --- |
| Hb A | 60 | 52 | 61 | 34 | 54 | 27 | 37 | 57 | 56 | 55 |
| Hb A2/CE | 0 | 0 | 0 | 0 | 0 | 16 | 0 | 0 | 0 | 0 |
| Hb F | 0 | 0 | 0 | 35 | 0 | 28 | 35 | 0 | 0 | 0 |
| Hb S | 40 | 48 | 39 | 31 | 46 | 29 | 28 | 43 | 44 | 45 |
| Total | 100 | 100 | 100 | 100 | 100 | 100 | 100 | 100 | 100 | 100 |

**B. Sickle-Chex Negative**

| **Gazelle band** | **Run 1** | **Run 2** | **Run 3** | **Run 4** | **Run 5** | **Run 6** | **Run 7** | **Run 8** | **Run 9** | **Run 10** |
| --- | --- | --- | --- | --- | --- | --- | --- | --- | --- | --- |
| Hb A | 67 | 57 | 61 | 62 | 66 | 66 | 57 | 62 | 58 | 62 |
| Hb A2/CE | 0 | 0 | 0 | 0 | 0 | 0 | 0 | 0 | 0 | 0 |
| Hb F | 33 | 43 | 39 | 38 | 34 | 34 | 43 | 38 | 42 | 38 |
| Hb S | 0 | 0 | 0 | 0 | 0 | 0 | 0 | 0 | 0 | 0 |
| Total | 100 | 100 | 100 | 100 | 100 | 100 | 100 | 100 | 100 | 100 |

**C. Bio-Rad Level 1**

| **Gazelle band** | **Run 1** | **Run 2** | **Run 3** | **Run 4** | **Run 5** | **Run 6** | **Run 7** | **Run 8** | **Run 9** | **Run 10** |
| --- | --- | --- | --- | --- | --- | --- | --- | --- | --- | --- |
| Hb A | 73 | 97 | 20 | 77 | 71 | 97 | 100 | 73 | 100 | 67 |
| Hb A2/CE | 0 | 3 | 0 | 3 | 3 | 3 | 0 | 0 | 0 | 0 |
| Hb F | 27 | 0 | 69 | 20 | 26 | 0 | 0 | 27 | 0 | 33 |
| Hb S | 0 | 0 | 11 | 0 | 0 | 0 | 0 | 0 | 0 | 0 |
| Total | 100 | 100 | 100 | 100 | 100 | 100 | 100 | 100 | 100 | 100 |

**D. Bio-Rad Level 2**

| **Gazelle band** | **Run 1** | **Run 2** | **Run 3** | **Run 4** | **Run 5** | **Run 6** | **Run 7** | **Run 8** | **Run 9** | **Run 10** |
| --- | --- | --- | --- | --- | --- | --- | --- | --- | --- | --- |
| Hb A | 39 | 54 | 61 | 59 | 60 | 60 | 35 | 61 | 53 | 60 |
| Hb A2/CE | 0 | 0 | 0 | 0 | 0 | 0 | 0 | 0 | 0 | 0 |
| Hb F | 38 | 0 | 0 | 0 | 0 | 0 | 32 | 0 | 0 | 0 |
| Hb S | 23 | 46 | 39 | 41 | 40 | 40 | 33 | 39 | 47 | 40 |
| Total | 100 | 100 | 100 | 100 | 100 | 100 | 100 | 100 | 100 | 100 |

Abbreviations: Hb, hemoglobin; Hb A2/CE, combined analytical region containing Hb A2, Hb C, and Hb E.

Interpretive note: Intermittent reporting of Hb A2/CE, Hb F, or Hb S in some commercial control materials resulted in high or noninterpretable CV estimates. These raw results are provided to demonstrate the run-to-run pattern of band assignment and to allow independent evaluation of the summary precision statistics.

**Supplementary Table S3.** Raw Gazelle hemoglobin-fraction results for residual patient-control samples.

The normal and Hb SC residual patient-control samples were each analyzed in duplicate over five study days, providing 10 measurements per sample. Results represent the percentage of total hemoglobin assigned by the Gazelle device to the Hb A, Hb A2/CE, Hb F, and Hb S bands. A value of 0 indicates that the device did not report a measurable band in that region and should not be interpreted as an independently confirmed concentration of absolute zero. All replicate results are shown, and no results were excluded as outliers.

**A. Normal patient-control sample**

| **Study day** | **Replicate** | **Hb A (%)** | **Hb A2/CE (%)** | **Hb F (%)** | **Hb S (%)** | **Total (%)** |
| --- | --- | --- | --- | --- | --- | --- |
| 1 | 1 | 97 | 3 | 0 | 0 | 100 |
| 1 | 2 | 97 | 3 | 0 | 0 | 100 |
| 2 | 1 | 97 | 3 | 0 | 0 | 100 |
| 2 | 2 | 97 | 3 | 0 | 0 | 100 |
| 3 | 1 | 97 | 3 | 0 | 0 | 100 |
| 3 | 2 | 97 | 3 | 0 | 0 | 100 |
| 4 | 1 | 97 | 3 | 0 | 0 | 100 |
| 4 | 2 | 98 | 2 | 0 | 0 | 100 |
| 5 | 1 | 97 | 3 | 0 | 0 | 100 |
| 5 | 2 | 97 | 3 | 0 | 0 | 100 |

**B. Hb SC patient-control sample**

| **Study day** | **Replicate** | **Hb A (%)** | **Hb A2/CE (%)** | **Hb F (%)** | **Hb S (%)** | **Total (%)** |
| --- | --- | --- | --- | --- | --- | --- |
| 1 | 1 | 0 | 58 | 4 | 38 | 100 |
| 1 | 2 | 0 | 60 | 0 | 40 | 100 |
| 2 | 1 | 0 | 62 | 0 | 38 | 100 |
| 2 | 2 | 0 | 56 | 0 | 44 | 100 |
| 3 | 1 | 0 | 60 | 0 | 40 | 100 |
| 3 | 2 | 0 | 61 | 0 | 39 | 100 |
| 4 | 1 | 0 | 54 | 0 | 46 | 100 |
| 4 | 2 | 0 | 53 | 4 | 43 | 100 |
| 5 | 1 | 0 | 55 | 4 | 41 | 100 |
| 5 | 2 | 0 | 54 | 4 | 42 | 100 |

Abbreviations: Hb, hemoglobin; Hb A2/CE, combined analytical region containing Hb A2, Hb C, and Hb E.

Interpretive note: In the Hb SC patient-control sample, the fraction reported in the Hb A2/CE region predominantly represents Hb C rather than Hb A2. Hb F was reported in only 4 of 10 measurements of the Hb SC sample, each at 4%, indicating intermittent detection at a low concentration. The normal patient-control sample demonstrated consistent reporting of Hb A and Hb A2/CE across all measurements.

**Supplementary Table S4.** Calculated expected concentrations and duplicate Gazelle results for mixing study 1.

Study 1 mixed a normal specimen (Level 1) with a high-Hb S specimen (Level 5).

**Panel A. Mixing design and observed Gazelle results**

| **Hb fraction** | **Level** | **Mixing ratio L1:L5** | **Expected (%)** | **Replicate 1 (%)** | **Replicate 2 (%)** | **Mean observed (%)** |
| --- | --- | --- | --- | --- | --- | --- |
| Hb A2/CE | 1 | 100:0 | 3.000 | 3.000 | 3.000 | 3.000 |
| Hb A2/CE | 2 | 75:25 | 2.250 | 0.000 | 0.000 | 0.000 |
| Hb A2/CE | 3 | 50:50 | 1.500 | 0.000 | 0.000 | 0.000 |
| Hb A2/CE | 4 | 25:75 | 0.750 | 0.000 | 0.000 | 0.000 |
| Hb A2/CE | 5 | 0:100 | 0.000 | 0.000 | 0.000 | 0.000 |
| Hb F | 1 | 100:0 | 0.000 | 0.000 | 0.000 | 0.000 |
| Hb F | 2 | 75:25 | 4.250 | 0.000 | 0.000 | 0.000 |
| Hb F | 3 | 50:50 | 8.500 | 0.000 | 0.000 | 0.000 |
| Hb F | 4 | 25:75 | 12.750 | 13.000 | 13.000 | 13.000 |
| Hb F | 5 | 0:100 | 17.000 | 18.000 | 16.000 | 17.000 |
| Hb S | 1 | 100:0 | 0.000 | 0.000 | 0.000 | 0.000 |
| Hb S | 2 | 75:25 | 20.750 | 15.000 | 0.000 | 7.500 |
| Hb S | 3 | 50:50 | 41.500 | 37.000 | 46.000 | 41.500 |
| Hb S | 4 | 25:75 | 62.250 | 70.000 | 70.000 | 70.000 |
| Hb S | 5 | 0:100 | 83.000 | 82.000 | 84.000 | 83.000 |
| Hb A | 1 | 100:0 | 97.000 | 97.000 | 97.000 | 97.000 |
| Hb A | 2 | 75:25 | 72.750 | 86.000 | 100.000 | 93.000 |
| Hb A | 3 | 50:50 | 48.500 | 63.000 | 54.000 | 58.500 |
| Hb A | 4 | 25:75 | 24.250 | 17.000 | 19.000 | 18.000 |
| Hb A | 5 | 0:100 | 0.000 | 0.000 | 0.000 | 0.000 |

**Panel B. Deviation and regression results**

| **Hb fraction** | **Level** | **Difference from expected (pp)** | **Absolute deviation (pp)** | **Percent deviation** | **Fitted regression (%)** | **Regression residual (pp)** | **Notes** |
| --- | --- | --- | --- | --- | --- | --- | --- |
| Hb A2/CE | 1 | 0.000 | 0.000 | 0.0% | 1.800 | 1.200 | Unmixed L1 |
| Hb A2/CE | 2 | -2.250 | 2.250 | -100.0% | 1.200 | -1.200 | — |
| Hb A2/CE | 3 | -1.500 | 1.500 | -100.0% | 0.600 | -0.600 | — |
| Hb A2/CE | 4 | -0.750 | 0.750 | -100.0% | 0.000 | 0.000 | — |
| Hb A2/CE | 5 | 0.000 | 0.000 | — | -0.600 | 0.600 | Unmixed L5 |
| Hb F | 1 | 0.000 | 0.000 | — | -3.400 | 3.400 | Unmixed L1 |
| Hb F | 2 | -4.250 | 4.250 | -100.0% | 1.300 | -1.300 | — |
| Hb F | 3 | -8.500 | 8.500 | -100.0% | 6.000 | -6.000 | — |
| Hb F | 4 | 0.250 | 0.250 | 2.0% | 10.700 | 2.300 | — |
| Hb F | 5 | 0.000 | 0.000 | 0.0% | 15.400 | 1.600 | Unmixed L5 |
| Hb S | 1 | 0.000 | 0.000 | — | -5.300 | 5.300 | Unmixed L1 |
| Hb S | 2 | -13.250 | 13.250 | -63.9% | 17.550 | -10.050 | — |
| Hb S | 3 | 0.000 | 0.000 | 0.0% | 40.400 | 1.100 | — |
| Hb S | 4 | 7.750 | 7.750 | 12.4% | 63.250 | 6.750 | — |
| Hb S | 5 | 0.000 | 0.000 | 0.0% | 86.100 | -3.100 | Unmixed L5 |
| Hb A | 1 | 0.000 | 0.000 | 0.0% | 107.100 | -10.100 | Unmixed L1 |
| Hb A | 2 | 20.250 | 20.250 | 27.8% | 80.200 | 12.800 | — |
| Hb A | 3 | 10.000 | 10.000 | 20.6% | 53.300 | 5.200 | — |
| Hb A | 4 | -6.250 | 6.250 | -25.8% | 26.400 | -8.400 | — |
| Hb A | 5 | 0.000 | 0.000 | — | -0.500 | 0.500 | Unmixed L5 |

Abbreviations: Hb, hemoglobin; Hb A2/CE, combined analytical region containing Hb A2, Hb C, and Hb E; L1, Level 1; L5, Level 5; pp, percentage points. Unmixed L1/L5 indicates the corresponding unmixed endpoint specimen.

Expected concentrations were proportionally interpolated from the two unmixed endpoints. Difference = mean observed − expected; absolute deviation = |difference|; percent deviation was not calculated when expected = 0%; residual = mean observed − fitted regression value. Gazelle 0% indicates no measurable band was reported and does not independently confirm an absolute concentration of zero.

**Supplementary Table S5.** Calculated expected concentrations and duplicate Gazelle results for mixing study 2.

Study 2 mixed a high-Hb A2/CE–Hb F specimen (Level 1) with a predominantly normal specimen (Level 5).

**Panel A. Mixing design and observed Gazelle results**

| **Hb fraction** | **Level** | **Mixing ratio L1:L5** | **Expected (%)** | **Replicate 1 (%)** | **Replicate 2 (%)** | **Mean observed (%)** |
| --- | --- | --- | --- | --- | --- | --- |
| Hb A2/CE | 1 | 100:0 | 75.500 | 78.000 | 73.000 | 75.500 |
| Hb A2/CE | 2 | 75:25 | 57.375 | 55.000 | 56.000 | 55.500 |
| Hb A2/CE | 3 | 50:50 | 39.250 | 9.000 | 21.000 | 15.000 |
| Hb A2/CE | 4 | 25:75 | 21.125 | 9.000 | 9.000 | 9.000 |
| Hb A2/CE | 5 | 0:100 | 3.000 | 3.000 | 3.000 | 3.000 |
| Hb F | 1 | 100:0 | 24.500 | 22.000 | 27.000 | 24.500 |
| Hb F | 2 | 75:25 | 18.375 | 18.000 | 15.000 | 16.500 |
| Hb F | 3 | 50:50 | 12.250 | 20.000 | 18.000 | 19.000 |
| Hb F | 4 | 25:75 | 6.125 | 0.000 | 0.000 | 0.000 |
| Hb F | 5 | 0:100 | 0.000 | 0.000 | 0.000 | 0.000 |
| Hb S | 1 | 100:0 | 0.000 | 0.000 | 0.000 | 0.000 |
| Hb S | 2 | 75:25 | 0.000 | 0.000 | 0.000 | 0.000 |
| Hb S | 3 | 50:50 | 0.000 | 0.000 | 0.000 | 0.000 |
| Hb S | 4 | 25:75 | 0.000 | 0.000 | 0.000 | 0.000 |
| Hb S | 5 | 0:100 | 0.000 | 0.000 | 0.000 | 0.000 |
| Hb A | 1 | 100:0 | 0.000 | 0.000 | 0.000 | 0.000 |
| Hb A | 2 | 75:25 | 24.250 | 27.000 | 29.000 | 28.000 |
| Hb A | 3 | 50:50 | 48.500 | 71.000 | 61.000 | 66.000 |
| Hb A | 4 | 25:75 | 72.750 | 91.000 | 97.000 | 94.000 |
| Hb A | 5 | 0:100 | 97.000 | 97.000 | 97.000 | 97.000 |

**Panel B. Deviation and regression results**

| **Hb fraction** | **Level** | **Difference from expected (pp)** | **Absolute deviation (pp)** | **Percent deviation** | **Fitted regression (%)** | **Regression residual (pp)** | **Notes** |
| --- | --- | --- | --- | --- | --- | --- | --- |
| Hb A2/CE | 1 | 0.000 | 0.000 | 0.0% | 69.900 | 5.600 | Unmixed L1 |
| Hb A2/CE | 2 | -1.875 | 1.875 | -3.3% | 50.750 | 4.750 | — |
| Hb A2/CE | 3 | -24.250 | 24.250 | -61.8% | 31.600 | -16.600 | — |
| Hb A2/CE | 4 | -12.125 | 12.125 | -57.4% | 12.450 | -3.450 | — |
| Hb A2/CE | 5 | 0.000 | 0.000 | 0.0% | -6.700 | 9.700 | Unmixed L5 |
| Hb F | 1 | 0.000 | 0.000 | 0.0% | 25.100 | -0.600 | Unmixed L1 |
| Hb F | 2 | -1.875 | 1.875 | -10.2% | 18.550 | -2.050 | — |
| Hb F | 3 | 6.750 | 6.750 | 55.1% | 12.000 | 7.000 | — |
| Hb F | 4 | -6.125 | 6.125 | -100.0% | 5.450 | -5.450 | — |
| Hb F | 5 | 0.000 | 0.000 | — | -1.100 | 1.100 | Unmixed L5 |
| Hb S | 1 | 0.000 | 0.000 | — | — | — | Unmixed L1 |
| Hb S | 2 | 0.000 | 0.000 | — | — | — | — |
| Hb S | 3 | 0.000 | 0.000 | — | — | — | — |
| Hb S | 4 | 0.000 | 0.000 | — | — | — | — |
| Hb S | 5 | 0.000 | 0.000 | — | — | — | Unmixed L5 |
| Hb A | 1 | 0.000 | 0.000 | — | 5.000 | -5.000 | Unmixed L1 |
| Hb A | 2 | 3.750 | 3.750 | 15.5% | 31.000 | -3.000 | — |
| Hb A | 3 | 17.500 | 17.500 | 36.1% | 57.000 | 9.000 | — |
| Hb A | 4 | 21.250 | 21.250 | 29.2% | 83.000 | 11.000 | — |
| Hb A | 5 | 0.000 | 0.000 | 0.0% | 109.000 | -12.000 | Unmixed L5 |

Abbreviations: Hb, hemoglobin; Hb A2/CE, combined analytical region containing Hb A2, Hb C, and Hb E; L1, Level 1; L5, Level 5; pp, percentage points. Unmixed L1/L5 indicates the corresponding unmixed endpoint specimen.

Expected concentrations were proportionally interpolated from the two unmixed endpoints. Difference = mean observed − expected; absolute deviation = |difference|; percent deviation was not calculated when expected = 0%; residual = mean observed − fitted regression value. Gazelle 0% indicates no measurable band was reported and does not independently confirm an absolute concentration of zero.

**Supplementary Table S6.** Demographic summary of method-comparison study samples.

| **Characteristic** | **Summary** |
| --- | --- |
| Total participants | 46 |
| Age, mean ± SD | 7.7 ± 10.6 years |
| Age, median (IQR) | 1.5 years (0.44–14.5) |
| Age range | 0.107–48 years |
| Pediatric participants, <18 years | 41 (89.1%) |
| Adult participants, ≥18 years | 5 (10.9%) |
| Female | 17 (37.0%) |
| Male | 29 (63.0%) |

**Supplementary Table S7.** Age-group distribution of method-comparison study samples.

| **Age group** | **n (%)** |
| --- | --- |
| <1 year | 18 (39.1%) |
| 1–<5 years | 8 (17.4%) |
| 5–<12 years | 7 (15.2%) |
| 12–<18 years | 8 (17.4%) |
| ≥18 years | 5 (10.9%) |
| **Total** | **46 (100%)** |

**Supplementary Table S8.** Capillarys phenotype distribution. Normal and beta-thalassemia-associated classifications are grouped; related subtype findings are grouped by the major Capillarys phenotype.

| **Capillarys phenotype** | **n** | **%** |
| --- | --- | --- |
| Normal | 16 | 34.8% |
| ASF | 5 | 10.9% |
| AF or AF with secondary variant | 4 | 8.7% |
| Beta thalassemia | 4 | 8.7% |
| AS | 3 | 6.5% |
| Alpha thalassemia or iron deficiency pattern | 3 | 6.5% |
| SF/sickle–β-thalassemia | 3 | 6.5% |
| FAS | 2 | 4.3% |
| SAF | 2 | 4.3% |
| Other hemoglobin variant with Hb F (Hb Inkster, Hb Toulon) | 2 | 4.3% |
| FAC/Hb C trait | 1 | 2.2% |
| FCA/Hb C–β⁺-thalassemia | 1 | 2.2% |
| **Total** | **46** | **100%** |

**Supplementary Table S9.** Gazelle phenotype distribution.

| **Gazelle phenotype** | **n** | **%** |
| --- | --- | --- |
| Normal | 21 | 45.7% |
| ASF | 6 | 13.0% |
| AF | 4 | 8.7% |
| Beta thalassemia | 3 | 6.5% |
| SF | 3 | 6.5% |
| AS | 2 | 4.3% |
| FAS | 2 | 4.3% |
| SAF | 2 | 4.3% |
| AC | 1 | 2.2% |
| FAC | 1 | 2.2% |
| FC | 1 | 2.2% |
| **Total** | **46** | **100%** |

**Supplementary Table S10.** Paired phenotype results and concordance classification for Gazelle and Capillarys.

| **Sample** | **Age, y** | **Gazelle phenotype** | **Capillarys phenotype** | **Concordance classification** |
| --- | --- | --- | --- | --- |
| 1 | 11 | AC | AS | Material discordance |
| 2 | 2 | Normal | Normal | Exact |
| 3 | 0.167 | FAC | FAC–C trait | Clinically harmonized |
| 4 | 1 | AS | ASF | Clinically harmonized |
| 5 | 15 | Normal | Normal | Exact |
| 6 | 0.25 | AF | AF + Hb Bart’s–alpha thalassemia | Secondary discordance |
| 7 | 15 | Normal | A + decreased A2–alpha thalassemia or iron deficiency | Material discordance |
| 8 | 0.33 | AF | AF + gamma fetal variant | Secondary discordance |
| 9 | 0.833 | Normal | AF + normal | Clinically harmonized |
| 10 | 8 | Beta thal | A + increased A2–beta thalassemia | Clinically harmonized |
| 11 | 19 | Normal | AF + elevated F | Material discordance |
| 12 | 0.167 | FC | FCA–HbC beta-plus thalassemia | Secondary discordance |
| 13 | 5 | Beta thal | A + increased A2–beta thalassemia | Clinically harmonized |
| 14 | 0.25 | ASF | ASF | Exact |
| 15 | 17 | Normal | Normal | Exact |
| 16 | 0.833 | ASF | AS–normal F | Clinically harmonized |
| 17 | 0.583 | Normal | Normal-AF | Clinically harmonized |
| 18 | 22 | Normal | Normal | Exact |
| 19 | 0.667 | SF | SF–sickle/beta thalassemia | Clinically harmonized |
| 20 | 10 | Normal | Normal | Exact |
| 21 | 16 | Normal | A–decreased A2–alpha thalassemia or iron deficiency | Material discordance |
| 22 | 0.107 | FAS | FAS | Exact |
| 23 | 1 | A2 increased–beta thal | A–increased A2–beta thalassemia | Clinically harmonized |
| 24 | 2 | Normal | Normal | Exact |
| 25 | 8 | SAF | SAF | Exact |
| 26 | 15 | Normal | A–A2 decreased–alpha thalassemia or iron deficiency | Material discordance |
| 27 | 1 | Normal | Normal | Exact |
| 28 | 0.5 | AF | AF + gamma variant | Secondary discordance |
| 29 | 16 | Normal | Normal | Exact |
| 30 | 8 | Normal | Normal | Exact |
| 31 | 0.167 | AF | Normal-AF | Clinically harmonized |
| 32 | 34 | Normal | Normal | Exact |
| 33 | 0.167 | AF | A + Hb Toulon with F | Secondary discordance |
| 34 | 32 | Normal | Normal | Exact |
| 35 | 3 | Normal | Normal | Exact |
| 36 | 7 | Normal | Normal | Exact |
| 37 | 0.5 | ASF | ASF | Exact |
| 38 | 17 | SF | SF–sickle/beta thalassemia | Clinically harmonized |
| 39 | 13 | SAF | SAF | Exact |
| 40 | 0.417 | ASF | ASF | Exact |
| 41 | 1 | AS | AS–A2 mildly increased | Secondary discordance |
| 42 | 0.167 | FAS | FAS | Exact |
| 43 | 0.167 | ASF | ASF | Exact |
| 44 | 0.25 | ASF | A + Hb Inkster with F | Material discordance |
| 45 | 1 | Normal, no F | AF + increased A2–beta thalassemia | Material discordance |
| 46 | 48 | SF | SF–sickle/beta thalassemia | Clinically harmonized |

Exact agreement required identical phenotype reporting. Clinically harmonized agreement allowed differences in nomenclature or reporting of age-appropriate Hb F that did not alter the major phenotype. Secondary discordance indicated agreement in the primary hemoglobin pattern with an additional finding identified by Capillarys but not characterized by Gazelle. Material discordance indicated disagreement involving the principal hemoglobin phenotype or classification as an age-appropriate normal versus potentially abnormal pattern.

**Supplementary Table S11.** Secondary and materially discordant phenotype results between Gazelle and Capillarys.

**A. Primary-pattern concordant with secondary discordance**

| **Sample** | **Age, y** | **Gazelle phenotype** | **Capillarys phenotype** | **Secondary finding** |
| --- | --- | --- | --- | --- |
| 6 | 0.25 | AF | AF + Hb Bart’s–alpha thalassemia | Hb Bart’s not identified |
| 8 | 0.33 | AF | AF + gamma fetal variant | Gamma-chain variant not identified |
| 12 | 0.167 | FC | FCA–HbC beta-plus thalassemia | Hb A/beta-plus thalassemia subtype not fully characterized |
| 28 | 0.5 | AF | AF + gamma variant | Gamma-chain variant not identified |
| 33 | 0.167 | AF | A + Hb Toulon with F | Hb Toulon not identified |
| 41 | 1 | AS | AS–A2 mildly increased | Mildly increased Hb A2 not identified |

**B. Materially discordant results**

| **Sample** | **Age, y** | **Gazelle phenotype** | **Capillarys phenotype** | **Nature of discordance** |
| --- | --- | --- | --- | --- |
| 1 | 11 | AC | AS | Hb C-containing versus Hb S-containing phenotype |
| 7 | 15 | Normal | A + decreased A2–alpha thalassemia or iron deficiency | Potential abnormality reported as normal |
| 11 | 19 | Normal | AF + elevated F | Elevated Hb F not identified |
| 21 | 16 | Normal | A–decreased A2–alpha thalassemia or iron deficiency | Potential abnormality reported as normal |
| 26 | 15 | Normal | A–A2 decreased–alpha thalassemia or iron deficiency | Potential abnormality reported as normal |
| 44 | 0.25 | ASF | A + Hb Inkster with F | Uncommon variant interpreted as an Hb S-containing pattern |
| 45 | 1 | Normal, no F | AF + increased A2–beta thalassemia | Elevated Hb F/Hb A2 beta-thalassemia pattern not identified |

**Supplementary Table S12.** Age-appropriate binary classification performance of Gazelle relative to Capillarys.

**A. 2 × 2 classification table**

|  | **Capillarys abnormal** | **Capillarys age-appropriate normal** | **Total** |
| --- | --- | --- | --- |
| Gazelle abnormal | 21 | 0 | 21 |
| Gazelle age-appropriate normal | 9 | 16 | 25 |
| **Total** | **30** | **16** | **46** |

**B. Diagnostic performance estimates**

| **Measure** | **Estimate** | **95% CI** |
| --- | --- | --- |
| Sensitivity | 70.0% (21/30) | 52.1–83.3% |
| Specificity | 100% (16/16) | 80.6–100% |
| Positive predictive value | 100% (21/21) | 84.5–100% |
| Negative predictive value | 64.0% (16/25) | 44.5–79.8% |
| Overall accuracy | 80.4% (37/46) | 66.8–89.3% |

Reference-positive and reference-negative classifications were defined using age-appropriate phenotype interpretation, with separate consideration of patients ≤6 months and >6 months of age. An abnormal result was defined as a phenotype containing a hemoglobin variant or other potentially abnormal finding by Capillarys; age-appropriate fetal hemoglobin patterns in infants were not considered abnormal in isolation. Capillarys was used as the comparator method. Ninety-five percent confidence intervals were calculated using the Wilson method. PPV and NPV are specific to this enriched study cohort and should not be generalized to a screening population. Because decreased Hb A2 may reflect either alpha thalassemia or iron deficiency, this analysis reflects detection of an abnormal hemoglobin pattern rather than definitive diagnosis of a hemoglobinopathy.

**Supplementary Table S13.** Paired Hb F results for specimens in which Hb F was detected by Capillarys but reported as 0% by Gazelle.

| **Complete-pair ID** | **Capillarys Hb F, %** | **Gazelle Hb F, %** | **Difference (Gazelle − Capillarys), percentage points** |
| --- | --- | --- | --- |
| 8 | 1.2 | 0.0 | −1.2 |
| 9 | 3.1 | 0.0 | −3.1 |
| 12 | 11.4 | 0.0 | −11.4 |
| 13 | 1.1 | 0.0 | −1.1 |
| 14 | 9.2 | 0.0 | −9.2 |
| 16 | 0.4 | 0.0 | −0.4 |
| 17 | 2.9 | 0.0 | −2.9 |
| 21 | 0.8 | 0.0 | −0.8 |
| 22 | 1.1 | 0.0 | −1.1 |
| 24 | 0.9 | 0.0 | −0.9 |
| 27 | 0.5 | 0.0 | −0.5 |
| 31 | 0.5 | 0.0 | −0.5 |
| 32 | 6.8 | 0.0 | −6.8 |

Hb F was measurable by Capillarys but reported as 0% by Gazelle in 13 of 32 complete numeric pairs. Ten of these specimens had Capillarys Hb F concentrations of 0.4%–3.1%; three had concentrations of 6.8%, 9.2%, and 11.4%. Thus, low Hb F concentration alone did not account for all Gazelle nondetections. Complete-pair IDs refer to the 32 specimens with paired numeric Hb F results used in the quantitative Hb F analysis.

**Supplementary Table S14.** Deming regression and Bland–Altman analysis of hemoglobin fractions, with and without uncommon variants.

| **Fraction** | **Analysis** | **n** | **Deming slope** | **Intercept** | **Mean bias, percentage points** | **95% limits of agreement** |
| --- | --- | --- | --- | --- | --- | --- |
| Hb A | All samples | 46 | 1.064 | −4.395 | +0.15 | −9.94 to +10.25 |
|  | Excluding uncommon variants | 42 | 1.056 | −4.051 | −0.10 | −9.47 to +9.27 |
| Hb F | All complete pairs | 32 | 0.999 | +1.040 | +1.03 | −8.70 to +10.76 |
|  | Excluding uncommon variants | 28 | 0.998 | +1.073 | +1.05 | −7.97 to +10.06 |
| Hb A2/CE | All complete numeric pairs | 43 | 1.182 | −1.351 | −0.72 | −4.56 to +3.11 |
|  | Excluding uncommon variants | 40 | 1.176 | −1.289 | −0.66 | −4.61 to +3.29 |
| Hb S | All complete pairs | 15 | 1.121 | −2.794 | +2.15 | −8.65 to +12.96 |
|  | Excluding uncommon variants | 15 | 1.121 | −2.794 | +2.15 | −8.65 to +12.96 |

Differences for Bland–Altman analysis were calculated as Gazelle minus Capillarys. Uncommon-variant exclusions included specimens containing Hb Bart’s, Hb Inkster, or gamma-chain variants. Blank or nonnumeric results were treated as missing and were not imputed as zero.

**Supplementary Table S15.** Sensitivity analysis of method-comparison results after exclusion of uncommon hemoglobin variants.

| **Fraction** | **Change in slope** | **Change in intercept** | **Change in mean bias** | **Interpretation** |
| --- | --- | --- | --- | --- |
| Hb A | −0.008 | +0.343 | −0.25 percentage points | Minimal change; limits narrowed slightly |
| Hb F | −0.001 | +0.033 | +0.02 percentage points | Essentially unchanged |
| Hb A2/CE | −0.006 | +0.062 | +0.07 percentage points | Essentially unchanged; negative bias persisted |
| Hb S | 0 | 0 | 0 | No uncommon-variant specimen had a complete Hb S pair; analysis unchanged |

**Supplementary Table S16.** Complete paired phenotype and quantitative hemoglobin fraction results obtained by Sebia Capillarys and Gazelle.

**A. Capillarys phenotype and quantitative fractions**

| **Sample** | **Age, y** | **Gazelle phenotype** | **Capillarys phenotype** | **Hb A** | **Hb F** | **Hb A2** | **Hb S** | **Hb C** | **Hb E** | **Hb Bart’s** | **Hb I Texas** | **Other** | **A2/C/E** |
| --- | --- | --- | --- | --- | --- | --- | --- | --- | --- | --- | --- | --- | --- |
| 1 | 11 | AC | AS | 86.3 | 0.0 | 2.4 | 11.3 | 0.0 | NR | NR | NR | NR | 2.4 |
| 2 | 2 | Normal | Normal | 96.4 | 1.2 | 2.4 | 0.0 | 0.0 | NR | NR | NR | NR | 2.4 |
| 3 | 0.167 | FAC | FAC–C trait | 21.6 | 65.8 | 0.0 | 0.0 | 12.6 | NR | NR | NR | NR | 12.6 |
| 4 | 1 | AS | ASF | 59.7 | 3.1 | 3.1 | 34.1 | 0.0 | NR | NR | NR | NR | 3.1 |
| 5 | 15 | Normal | Normal | 97.5 | 0.0 | 2.5 | NR | 0.0 | NR | NR | NR | NR | 2.5 |
| 6 | 0.25 | AF | AF + Hb Bart’s–alpha thalassemia | 76.9 | 20.1 | 1.7 | NR | 0.0 | NR | 1.3 | NR | NR | 1.7 |
| 7 | 15 | Normal | A + decreased A2–alpha thalassemia or iron deficiency | 98.0 | 0.0 | 2.0 | NR | 0.0 | NR | NR | NR | NR | 2.0 |
| 8 | 0.33 | AF | AF + gamma fetal variant | 85.0 | 9.8 | 1.8 | NR | 0.0 | NR | NR | NR | 3.4 | 1.8 |
| 9 | 0.833 | Normal | AF + normal | 86.4 | 11.4 | 2.2 | NR | NR | NR | NR | NR | NR | 2.2 |
| 10 | 8 | Beta thal | A + increased A2–beta thalassemia | 93.7 | 1.1 | 5.2 | NR | NR | NR | NR | NR | NR | 5.2 |
| 11 | 19 | Normal | AF + elevated F | 88.2 | 9.2 | 2.6 | NR | NR | NR | NR | NR | NR | 2.6 |
| 12 | 0.167 | FC | FCA–HbC beta-plus thalassemia | 6.0 | 66.1 | NR* | NR | 26.3 | NR | NR | NR | NR | 26.3 |
| 13 | 5 | Beta thal | A + increased A2–beta thalassemia | 94.7 | 0.0 | 5.3 | NR | NR | NR | NR | NR | NR | 5.3 |
| 14 | 0.25 | ASF | ASF | 46.0 | 18.7 | 2.5 | 32.8 | NR | NR | NR | NR | NR | 2.5 |
| 15 | 17 | Normal | Normal | 96.9 | 0.4 | 2.7 | NR | NR | NR | NR | NR | NR | 2.7 |
| 16 | 0.833 | ASF | AS–normal F | 55.2 | 2.2 | 3.2 | 39.4 | NR | NR | NR | NR | NR | 3.2 |
| 17 | 0.583 | Normal | Normal-AF | 94.4 | 2.9 | 2.7 | NR | NR | NR | NR | NR | NR | 2.7 |
| 18 | 22 | Normal | Normal | 97.0 | 0.0 | 3.0 | NR | NR | NR | NR | NR | NR | 3.0 |
| 19 | 0.667 | SF | SF–sickle/beta thalassemia | 0.0 | 38.0 | 1.9 | 60.1 | NR | NR | NR | NR | NR | 1.9 |
| 20 | 10 | Normal | Normal | 97.6 | 0.0 | 2.4 | NR | NR | NR | NR | NR | NR | 2.4 |
| 21 | 16 | Normal | A–decreased A2–alpha thalassemia or iron deficiency | 98.2 | NR | 1.9 | NR | NR | NR | NR | NR | NR | 1.9 |
| 22 | 0.107 | FAS | FAS | 24.5 | 63.0 | 0.7 | 11.8 | NR | NR | NR | NR | NR | 0.7 |
| 23 | 1 | A2 increased–beta thal | A–increased A2–beta thalassemia | 94.3 | 0.8 | 4.9 | NR | NR | NR | NR | NR | NR | 4.9 |
| 24 | 2 | Normal | Normal | 96.1 | 1.1 | 2.8 | NR | NR | NR | NR | NR | NR | 2.8 |
| 25 | 8 | SAF | SAF | 42.8 | 3.4 | 3.3 | 50.5 | NR | NR | NR | NR | NR | 3.3 |
| 26 | 15 | Normal | A–A2 decreased–alpha thalassemia or iron deficiency | 98.1 | 0.0 | 1.9 | NR | NR | NR | NR | NR | NR | 1.9 |
| 27 | 1 | Normal | Normal | 96.6 | 0.9 | 2.5 | NR | NR | NR | NR | NR | NR | 2.5 |
| 28 | 0.5 | AF | AF + gamma variant | 91.2 | 4.9 | 2.2 | NR | NR | NR | NR | NR | 1.7 | 2.2 |
| 29 | 16 | Normal | Normal | 97.3 | 0.0 | 2.7 | NR | NR | NR | NR | NR | NR | 2.7 |
| 30 | 8 | Normal | Normal | 97.3 | 0.0 | 2.7 | NR | NR | NR | NR | NR | NR | 2.7 |
| 31 | 0.167 | AF | Normal-AF | 72.7 | 25.6 | 1.7 | NR | NR | NR | NR | NR | NR | 1.7 |
| 32 | 34 | Normal | Normal | 96.8 | 0.5 | 2.7 | NR | NR | NR | NR | NR | NR | 2.7 |
| 33 | 0.167 | AF | A + Hb Toulon with F | 71.8 | 26.5 | 1.7 | NR | NR | NR | NR | NR | NR | 1.7 |
| 34 | 32 | Normal | Normal | 97.5 | 0.0 | 2.5 | NR | NR | NR | NR | NR | NR | 2.5 |
| 35 | 3 | Normal | Normal | 97.4 | 0.0 | 2.6 | NR | NR | NR | NR | NR | NR | 2.6 |
| 36 | 7 | Normal | Normal | 97.3 | 0.0 | 2.7 | NR | NR | NR | NR | NR | NR | 2.7 |
| 37 | 0.5 | ASF | ASF | 54.3 | 3.7 | 3.1 | 38.9 | NR | NR | NR | NR | NR | 3.1 |
| 38 | 17 | SF | SF–sickle/beta thalassemia | 0.0 | 15.5 | 3.4 | 81.1 | NR | NR | NR | NR | NR | 3.4 |
| 39 | 13 | SAF | SAF | 27.7 | 11.2 | 3.1 | 58.0 | NR | NR | NR | NR | NR | 3.1 |
| 40 | 0.417 | ASF | ASF | 56.0 | 4.3 | 3.1 | 36.6 | NR | NR | NR | NR | NR | 3.1 |
| 41 | 1 | AS | AS–A2 mildly increased | 56.9 | 0.5 | 3.4 | 39.2 | NR | NR | NR | NR | NR | 3.4 |
| 42 | 0.167 | FAS | FAS | 25.3 | 59.2 | 0.7 | 14.8 | NR | NR | NR | NR | NR | 0.7 |
| 43 | 0.167 | ASF | ASF | 45.0 | 28.4 | 2.2 | 24.4 | NR | NR | NR | NR | NR | 2.2 |
| 44 | 0.25 | ASF | A + Hb Inkster with F | 69.6 | 6.5 | 1.3 | NR | NR | NR | 19.0 | 3.1 | 0.5 | 1.3 |
| 45 | 1 | Normal, no F | AF + increased A2–beta thalassemia | 87.7 | 6.8 | 5.5 | NR | NR | NR | NR | NR | NR | 5.5 |
| 46 | 48 | SF | SF–sickle/beta thalassemia | 0.0 | 16.3 | 3.0 | 80.7 | NR | NR | NR | NR | NR | 3.0 |

**B. Gazelle quantitative fractions**

| **Sample** | **Age, y** | **Gazelle phenotype** | **Capillarys phenotype** | **Gazelle Hb A, %** | **Gazelle Hb F, %** | **Gazelle Hb A2/C/E, %** | **Gazelle Hb S, %** |
| --- | --- | --- | --- | --- | --- | --- | --- |
| 1 | 11 | AC | AS | 91.0 | 0.0 | 9.0 | 0.0 |
| 2 | 2 | Normal | Normal | 98.0 | 0.0 | 2.0 | NR |
| 3 | 0.167 | FAC | FAC–C trait | 20.0 | 68.0 | 12.0 | NR |
| 4 | 1 | AS | ASF | 67.0 | 0.0 | 0.0 | 33.0 |
| 5 | 15 | Normal | Normal | 98.0 | 0.0 | 2.0 | NR |
| 6 | 0.25 | AF | AF + Hb Bart’s–alpha thalassemia | 84.0 | 16.0 | 0.0 | NR |
| 7 | 15 | Normal | A + decreased A2–alpha thalassemia or iron deficiency | 97.0 | 0.0 | 3.0 | NR |
| 8 | 0.33 | AF | AF + gamma fetal variant | 95.0 | 5.0 | 0.0 | NR |
| 9 | 0.833 | Normal | AF + normal | 97.0 | NR | 3.0 | NR |
| 10 | 8 | Beta thal | A + increased A2–beta thalassemia | 95.0 | NR | 5.0 | NR |
| 11 | 19 | Normal | AF + elevated F | 97.0 | NR | 3.0 | NR |
| 12 | 0.167 | FC | FCA–HbC beta-plus thalassemia | 6.0 | 66.0 | 28.0 | NR |
| 13 | 5 | Beta thal | A + increased A2–beta thalassemia | 95.0 | NR | 5.0 | NR |
| 14 | 0.25 | ASF | ASF | 38.0 | 21.0 | 0.0 | 41.0 |
| 15 | 17 | Normal | Normal | 97.0 | 0.0 | 3.0 | NR |
| 16 | 0.833 | ASF | AS–normal F | 48.0 | 8.0 | 0.0 | 44.0 |
| 17 | 0.583 | Normal | Normal-AF | 97.0 | 0.0 | 3.0 | NR |
| 18 | 22 | Normal | Normal | 97.0 | 0.0 | 3.0 | NR |
| 19 | 0.667 | SF | SF–sickle/beta thalassemia | 0.0 | 30.0 | 0.0 | 70.0 |
| 20 | 10 | Normal | Normal | 98.0 | NR | 2.0 | NR |
| 21 | 16 | Normal | A–decreased A2–alpha thalassemia or iron deficiency | 97.0 | NR | 3.0 | NR |
| 22 | 0.107 | FAS | FAS | 21.0 | 61.0 | 0.0 | 18.0 |
| 23 | 1 | A2 increased–beta thal | A–increased A2–beta thalassemia | 95.0 | 0.0 | 5.0 | 0.0 |
| 24 | 2 | Normal | Normal | 97.0 | 0.0 | 3.0 | 0.0 |
| 25 | 8 | SAF | SAF | 30.0 | 11.0 | 0.0 | 59.0 |
| 26 | 15 | Normal | A–A2 decreased–alpha thalassemia or iron deficiency | 97.0 | NR | 3.0 | NR |
| 27 | 1 | Normal | Normal | 97.0 | NR | 3.0 | NR |
| 28 | 0.5 | AF | AF + gamma variant | 95.0 | 5.0 | NR | NR |
| 29 | 16 | Normal | Normal | 98.0 | NR | 2.0 | NR |
| 30 | 8 | Normal | Normal | 97.0 | NR | 3.0 | NR |
| 31 | 0.167 | AF | Normal-AF | 71.0 | 29.0 | 0.0 | NR |
| 32 | 34 | Normal | Normal | 97.0 | NR | 3.0 | NR |
| 33 | 0.167 | AF | A + Hb Toulon with F | 74.0 | 26.0 | 0.0 | NR |
| 34 | 32 | Normal | Normal | 97.0 | NR | 3.0 | NR |
| 35 | 3 | Normal | Normal | 97.0 | NR | 3.0 | NR |
| 36 | 7 | Normal | Normal | 97.0 | NR | 3.0 | NR |
| 37 | 0.5 | ASF | ASF | 49.0 | 11.0 | 0.0 | 40.0 |
| 38 | 17 | SF | SF–sickle/beta thalassemia | 0.0 | 16.0 | NR | 84.0 |
| 39 | 13 | SAF | SAF | 21.0 | 16.0 | 2.9 | 63.0 |
| 40 | 0.417 | ASF | ASF | 48.0 | 17.0 | 0.0 | 35.0 |
| 41 | 1 | AS | AS–A2 mildly increased | 63.0 | 0.0 | 0.0 | 37.0 |
| 42 | 0.167 | FAS | FAS | 24.0 | 58.0 | 0.0 | 18.0 |
| 43 | 0.167 | ASF | ASF | 40.0 | 39.0 | 0.0 | 21.0 |
| 44 | 0.25 | ASF | A + Hb Inkster with F | 60.0 | 19.0 | 0.0 | 21.0 |
| 45 | 1 | Normal, no F | AF + increased A2–beta thalassemia | 100.0 | 0.0 | 0.0 | NR |
| 46 | 48 | SF | SF–sickle/beta thalassemia | 0.0 | 17.0 | 0.0 | 83.0 |

Values are percentages of total hemoglobin. NR indicates that no numeric value was reported in the source dataset; explicit 0% values are retained as zero and should not be interpreted as independent confirmation that the fraction was analytically absent. Capillarys A2/C/E represents the composite comparator used for quantitative comparison with the combined Gazelle Hb A2/C/E region. For specimens containing Hb C or Hb E, the corresponding Capillarys fractions were incorporated into the composite value as applicable. Individual uncommon-variant fields reflect the source dataset and should be interpreted together with the final Capillarys phenotype assignment.
